# Investigating Achilles tendon adaptation to mechanical load: a computational model integrating collagen fibre orientation heterogeneity

**DOI:** 10.1007/s10237-025-02002-0

**Published:** 2025-08-24

**Authors:** Renate Janssen, Anna Gustafsson, Viktor Jönsson, Lorenzo Grassi, Maria Pierantoni, Hanna Isaksson

**Affiliations:** Department of Biomedical Engineering, Lund University, Box 118, 221 00 Lund, Sweden

**Keywords:** Collagen fibres, Synchrotron imaging, Poro-elasticity, Fibre-reinforced, Viscoelasticity, Finite element analysis

## Abstract

Tendons are known to adapt their structural and mechanical properties in response to mechanical loading, but the precise mechanisms underlying this adaptation remain poorly understood. A previous study on rat Achilles tendons compared the effect of unloading (Botox injections and orthosis) with free cage activity (full loading) and revealed that unloading impaired the mechanical response and resulted in more dispersed collagen fibre orientations. The current study investigates tendon mechanobiology by integrating this experimental fibre data into a finite element model. The aim is to evaluate whether the altered mechanical response after unloading results from changes in collagen fibre orientation, tendon geometry, or material properties. Collagen fibre orientation analysis was performed based on phase-contrast enhanced synchrotron X-ray tomography images. Two levels of collagen fibre orientation detail were implemented into the finite element model: 1) global fibre orientation analysis that averaged fibre directions across the entire tendon and 2) local orientation analysis that introduced spatial heterogeneity by incorporating element-specific fibre distributions. Our results indicate that the impaired mechanical response in unloaded tendons is mainly due to changes in fibre orientation distribution and geometry. The local collagen orientation analysis showed a lower overall force response, but did not alter the relative differences between fully loaded and unloaded tendons. Incorporating the increased heterogeneity may still be important for future studies of tendon mechanobiology. The established framework provides a robust tool for exploring tendon biomechanics, capturing detailed fibre information, and offering valuable insights into tendon adaptation under various conditions.

**Graphical abstract:**

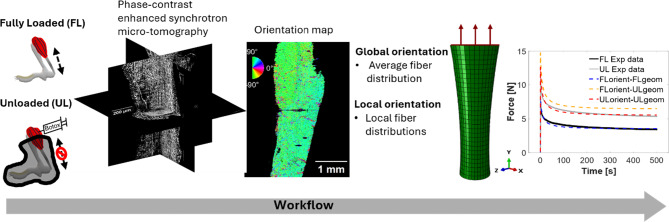

**Supplementary Information:**

The online version contains supplementary material available at 10.1007/s10237-025-02002-0.

## Introduction

Tendons transmit forces from muscles to bone, facilitating joint motion. Their structure consists of highly aligned collagen molecules, fibrils, fibres, and fibre bundles that lie mainly in the direction of the mechanical loads (Wang 2006). Tendons are mechanosensitive, meaning that they adapt their structure, composition, and mechanical properties to the mechanical loading (Wang 2006). However, the precise underlying mechanisms of tendon’s adaptation, and how it affects the tendon microstructure, collagen production, and mechanical performance remain poorly understood (Lavagnino et al. 2015). A better understanding of these processes is crucial for developing more effective strategies for treating and preventing tendon injuries (Notermans et al. 2019).

One way to deepen our understanding of tendon adaptation is by investigating the effects of unloading. Previous studies on humans found that Achilles tendon stiffness decreased by approximately 30% after 20 days of bed rest (Kubo et al. 2004), by about 60% following paralysis (Maganaris et al. 2006), and by 10% after 14 days of unilateral lower limb suspension (de Boer et al. 2007). Studying adaptation in human tendons is challenging due to high variability among individuals and ethical constraints to obtain biopsies from patients or volunteers (Snedeker and Foolen 2017). Thus, most data in the literature are derived from animal studies, which further allows for more precise control over the loading conditions (Lake et al. 2008). Different types of techniques are used to mimic unloading, such as cast immobilization, Botox treatment, and tail or hindlimb suspension (Notermans et al. 2021a). For example, Pierantoni et al. (2023) investigated the effect of unloading on rat Achilles tendons using Botox injections and an orthosis boot, a model earlier developed by Hammerman et al. (2018). The tendons were harvested and analysed at multiple length scales using synchrotron X-ray imaging and scattering techniques. The study revealed that unloading led to increased tendon length and cross-sectional area, accompanied by an impaired mechanical response characterized by a decreased elastic modulus and viscoelastic properties. Moreover, microstructural changes were visualized and quantified showing that the fibre orientations in the unloaded tendons were more dispersed than in the fully loaded tendons (Pierantoni et al. 2023).

To further improve the understanding of tendon biomechanics and mechanobiology, computational models can offer valuable insights by simulating the mechanical behaviour of different tissue components (Khayyeri et al. 2016; Notermans et al. 2019). Existing models range from simple to complex constitutive formulations (Thompson et al. 2017), with viscoelastic and hyperelastic models commonly employed to describe the matrix. Collagen fibres are often treated separately and introduced using e.g. transverse isotropic (Yin and Elliott 2004) or anisotropic constitutive formulations (Ciarletta et al. 2008; Kahn et al. 2010; Tang et al. 2011; Bajuri et al. 2016). To account for the important fluid component, Khayyeri et al. (2015) adapted a fibre-reinforced poro-viscoelastic model originally developed for cartilage (Wilson et al. 2005), using a uniform fibre orientation. While previous computational models have successfully captured key aspects of tendon mechanics, they rely on either simplified or a priori assumed fibre orientations, which limit their ability to represent the detailed microstructural adaptations of tendons under varying mechanical conditions. Although the study by Notermans et al. (2019) investigated how material parameters adapt to tendon unloading, it still assumed a single uniform fibre orientation. The recent evidence from Pierantoni et al. (2023) challenges this assumption and suggests that fibre orientations are not uniform and vary upon unloading. Since the main load-bearing component in tendons is the fibres, considering variation in their orientation could provide additional insights into tendon mechanical behaviour. Thus, the specific effects of unloading on fibre orientations and their contribution to tendon mechanics have not yet been explored.

This study therefore aims to gain a more comprehensive understanding of the tendon mechanics in response to unloading by advancing computational models with experimentally derived fibre orientation data. More specifically, we pose the question whether the fibre orientation alone can be responsible for the altered mechanical response between fully loaded and unloaded states found by Pierantoni et al. (2023), or whether material properties of the tendon also play a role. The answer to this question is obtained by combining the visco-hyper-poro-elastic model from Khayyeri et al. (2016), with detailed fibre orientations from the high-resolution imaging data of Pierantoni et al. (2023).

## Materials & methods

This study re-analyses phase-contrast enhanced synchrotron micro-tomography imaging data from Pierantoni et al. (2023) to obtain sample-specific spatial collagen fibre distributions in Achilles tendons from fully loaded and unloaded rats. The collagen fibre distributions were implemented into finite element (FE) models, and the force–time curves of the stress relaxation tests were compared to the experimental mechanical test data of Pierantoni et al. (2023).

**Fig. 1:**
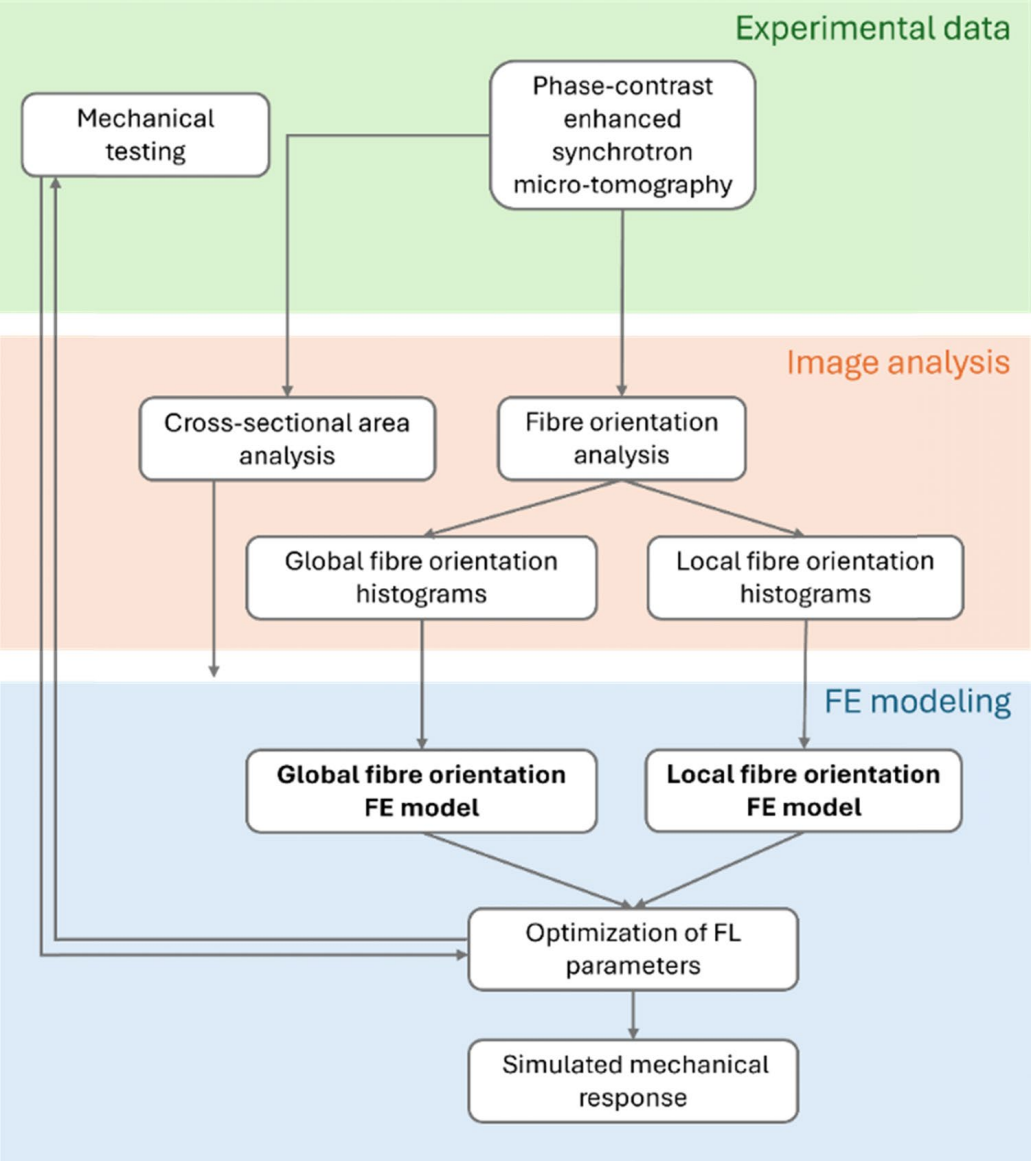
Methodological framework; Experimental data was acquired from Pierantoni et al. (2023). In this study, the image data was analysed to create the segmented geometry and perform orientation analysis for the global and local orientation cases. These fibre orientations were subsequently implemented into a finite element (FE) model to simulate the mechanical response. The FE model for the fully loaded (FL) case was specifically used for optimizing the material parameters

The collagen fibre distribution information was implemented into the FE model using two different approaches based on different levels of detail. First, the global fibre orientation model applied the same histograms representing the average orientation distribution of the entire tendon throughout the whole FE model. Secondly, the local fibre orientation model quantified the orientation distributions locally, creating element-specific histograms to capture spatial variation. These orientations were then mapped onto the FEM model. An overview of the approach is illustrated in Fig. 1.

### Experimental data on rat achilles tendon

The experimental data were obtained from high-resolution phase-contrast enhanced synchrotron micro-tomography data (Pierantoni et al. 2023). In the previous study, female Sprague–Dawley rats (age 10–12 weeks) were randomly divided into two groups for fully loading (FL, free cage activity) and unloading (UL*,* immobilization through Botox injections combined with a steel orthosis) and sacrificed after 4 weeks. Separate tendons were used for imaging and mechanical testing as high-resolution imaging may alter the mechanical properties. Please see supplementary material S1 for more details on both the imaging and the mechanical testing procedures.

**Fig. 2:**
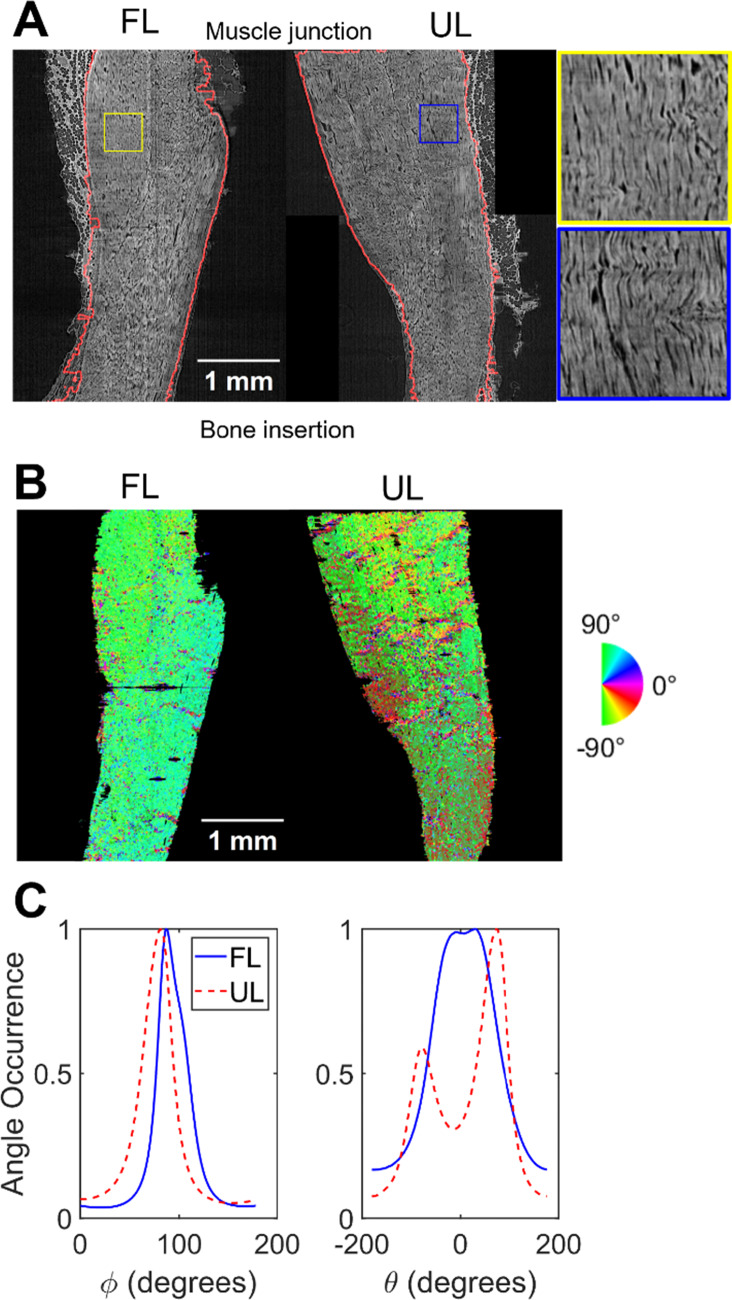
Image segmentation analysis. **A** Segmentation (red) of fully loaded (FL) and unloaded (UL) tendon, including zoom-ins to exemplify fiber structure. The zoom-ins are available in Supplementary movies 1–2. **B** Corresponding orientation maps of fibre organizations for the elevation angle. **C** Global azimuth (*θ*) and elevation (Ф) angle histograms for the FL and UL tendon

Pierantoni et al. (2023) presented a 3D orientation distribution based on a structure tensor approach (Krause et al. 2010; Saxena et al. 2019) on sub-volumes of the tendons. In the current study, we used the average mechanical response for each group (n = 9 for FL and n = 10 for UL) by averaging force values across all specimens at each timepoint. For imaging, we selected two representative tendons from the imaged groups (n = 7 for FL, n = 7 for UL): one from the FL and one from the UL group. These tendons were chosen based on their size, fibre orientation, and fibre density, ensuring that they reflect the characteristics of their respective groups (FL and UL). We then extended the 3D collagen fibre orientation analysis to capture the entire tendon. First, the original image volumes (voxel size 1.625 × 1.625 × 1.625 µm^3^) were isotropically downscaled with a factor 2 to 3.25 µm, resulting in images where structural details still were observed at the fibre scale (Pierantoni et al. 2023) (Fig. 2a**,** Supplementary movies 1–2). The images were then cropped to match the shortest captured tendon length of 4.25 mm (Fig. 2a), thus encompassing the entire free tendon length, but not including the muscle–tendon junction to closely mimic the mechanical testing setup. Thereafter, the images were binarized to separate the tendon from the background utilizing Otsu’s method (Otsu 1979), and adipose and mineralized tissue were removed through Ilastik’s pixel classification (Berg et al. 2019) combined with closing and area filtering techniques in MATLAB (R2024b, The MathWorks and Inc., 2024).

The pre-processed images (Fig. 2a) were used for calculating the cross-sectional areas along the tendon length and for the fibre orientation analysis. A second-order polynomial was fitted to the calculated areas to align the cross-sectional area of the FE model with the experimental cross-sectional area (Supplementary material S2).

Subsequently, the fibre orientation analysis from Pierantoni et al. (2023) was performed, where the data were first smoothed with a 3D Gaussian filter (*σ* = 1.5), and the structure tensor was also smoothed with a second 3D Gaussian filter (*ρ* = 1.5). Boundary effects of the orientation analysis were removed by another closing, area filtering, and dilation. The resulting fibre angles were represented as 3D orientation maps (Fig. 2b) from which a single orientation histogram for in-plane (azimuth, *θ*) and out-of-plane (elevation, φ) angles was created (Fig. 2c). These orientation histograms were used in the FE global orientation models, while the 3D orientation maps were used in the FE local orientation models.

### Constitutive model

The computational model used in this study is the transversely isotropic, fibre-reinforced visco-hyper-poro-elastic constitutive model by Khayyeri et al., (2016). The implementation as described by Notermans et al. (2019) was used and briefly summarized here.

The constitutive model consists of a non-fibrillar matrix part, describing the proteoglycan matrix of the tendon, and a fibrillar part describing the collagen fibres. The solid matrix is porous and fully saturated with water, resulting in the total stress being defined as:

1 $$\varvec{\sigma}_{total} = \varvec\sigma_{s} - p\varvec I = \varvec\sigma_{f} + \varvec\sigma_{m} - p\varvec I$$

where ${{\varvec{\upsigma}}}_{s}$ is the stress in the solid matrix; ${{\varvec{\upsigma}}}_{f}$ and ${{\varvec{\upsigma}}}_{m}$ the stresses in the collagen fibres and the non-fibrillar matrix, respectively. The fluid phase is incompressible and accounted for through the hydrostatic pressure *p*.

To capture the visco-elastic response of the collagen fibres, a standard linear solid model (Suplementary material S3) was used (Khayyeri et al. 2016). The elastic springs were modelled with an exponential stress–strain relationship (Wilson et al. 2005) (Eq. 2). Assuming that the fibres only carry load in tension, the Cauchy stress of a single fibre is given as:

2 $${\varvec{\sigma}}_{f} = \frac{\lambda }{J}{\varvec{P}}_{f} \vec{\user2{e}}_{f} \vec{\user2{e}}_{f}^{T} { }$$

3 $${\varvec{P}}_{f} = {\varvec{P}}_{1} + {\varvec{P}}_{2} = {\varvec{P}}_{1} + {\varvec{P}}_{\eta }$$

4 $${\varvec{P}}_{n} = \left\{ {\begin{array}{*{20}c} {E_{n} \left( {e^{{k_{n} \varepsilon_{n} }} - 1} \right)} & {\varepsilon_{n} > 0} \\ 0 & {\varepsilon_{n} \le 0} \\ \end{array} } \right.\;\;\;{\text{for }}n = 1,2$$

5 $${\varvec{P}}_{\eta } = \eta \dot{\varepsilon }_{\eta } = \eta \frac{{d\varepsilon_{\eta } }}{dt} = \eta \left( {\frac{{d\varepsilon_{1} }}{dt} - \frac{{d\varepsilon_{2} }}{dt}} \right) with \ \varepsilon_{1} = \varepsilon_{2} + \varepsilon_{\eta }$$

where $\lambda$ is the fibre stretch, $J$ the determinant of the deformation matrix ${\varvec{F}}$ and ${\overrightarrow{{\varvec{e}}}}_{f}$ a one-dimensional unit vector describing the current fibre orientation. ${{\varvec{P}}}_{1}$ and ${{\varvec{P}}}_{2}$ are the first Piola–Kirchhoff stresses of the spring in parallel and the maxwell component, respectively, and ${{\varvec{P}}}_{\eta }$ the first Piola–Kirchhoff stress of the dashpot. ${E}_{1}$ and ${k}_{1}$ are the stiffness parameters for the spring in parallel, ${E}_{2}$ and ${k}_{2}$ the stiffness parameters for the spring in series and $\eta$ the damping parameter. The strains of the respective springs are denoted as ${\varepsilon }_{1}$ and ${\varepsilon }_{2}$. Adding multiple fibres to the model results in:

6 $${\varvec{\sigma}}_{f,tot} = \mathop \sum \limits_{1}^{{n_{f} }} \frac{{\rho_{f,tot} }}{{n_{f} }}{\varvec{\sigma}}_{f}$$

where ${{\varvec{\sigma}}}_{f,\text{tot}}$ is the total fibre stress contribution, ${\text{n}}_{\text{f}}$ the total number of fibres, ${\rho }_{f,tot}$ the relative density of the fibres compared to the matrix and ${{\varvec{\sigma}}}_{f}$ the stress contribution of a single fibre (Eq. 2).

The matrix contribution is modelled as a transversely isotropic, hyperelastic St. Venant–Kirchhoff's strain energy function and the tendons permeability is void ratio dependent (Notermans et al. 2019).

### Finite element implementation

Cylindrical 3D geometries were created in Abaqus R2023x (Dassault Systèmes Simulia Corp., Providence, RI, USA) using the imaged tendon lengths (Fig. 2a), 4.25 mm and cross-sectional areas (Supplementary material S2). Eight-node trilinear displacement and pore pressure elements (C3D8P) were used with a maximum edge length 0.21 mm (3840 elements in total). Boundary conditions were applied using a cylindrical coordinate system, with the R-*θ* axis in the transversal plane and the Z-axis along the longitudinal direction of the tendon. A central node on the bottom surface was constrained in all directions, and the entire bottom surface was constrained in Z-direction. The top and bottom surfaces were constrained in *θ*-direction, and a zero-pore pressure was prescribed on the outer surface of the tendon.

To improve convergence of the FE analysis, the length of the tendon was increased by 10% at the bottom and top, thus reaching a total length of 5.32 mm. This extension allowed for a gradual transition of the fibre orientation towards ${\phi }$ = 90° at the actual image data. The gradual transition in fibre orientation was achieved by retrieving the Y-coordinates of the elements and modifying their angles as follows:

7 $${\phi }_{1} = 90 - \frac{T}{0.1h}\left( {90 - \phi_{0} } \right)$$

where ${\phi }_{1}$ and ${\phi }_{0}$ represent the new and original orientation, respectively, *h* is the total height of the tendon, and *T* is the Y-position of the element. More specifically, *T* is defined as the Y-coordinate for the bottom 10% and *h-Y* for the top 10%. Additionally, the cross-sectional area of the extended segments corresponded with the polynomial fitted to the area profile of the original tendon, ensuring consistency in cross-sectional geometry across the entire model.

The stress relaxation protocol described by Pierantoni et al. (2023) was implemented by applying a displacement equivalent to 8% strain to the top surface along the Z-direction at a speed of 1 mm/s, followed by a 500-s relaxation phase. The model's reaction forces were compared to the experimentally measured force.

###  Global fibre orientation

**Fig. 3:**
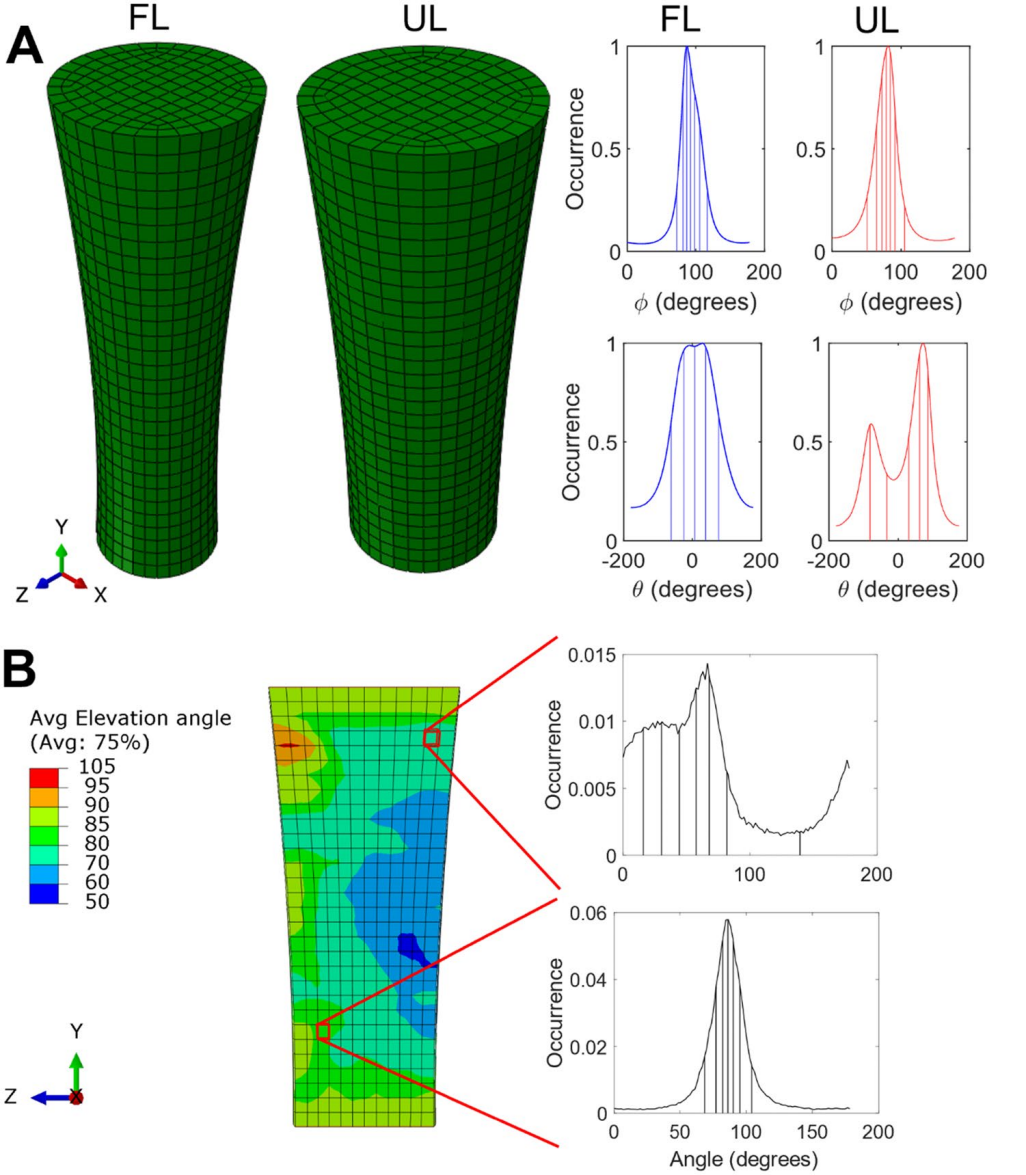
Global and local fibre orientation implementation in the fully loaded (FL) and unloaded (UL) tendon. **A** Global fibre orientation implementation, where all elements have the same fibre orientations. **B** Local fibre orientation implementation, where each element has its own fibre orientation. The image is exemplified based on the UL tendon

To implement the global fibre orientations into the FE model, a cumulative area plot was created from the single orientation histogram of the azimuth (φ) and elevation (*θ*) angle (Fig. 2c). The y-axis of the cumulative area plot was subsequently partitioned into (n + 1) equal intervals, where n represents the total number of fibres to be defined. For each of these intervals, the corresponding angles on the x-axis of the cumulative area plot were identified. These identified angles, representing the midpoints n-fibre distribution ranges, were then assigned as the fibre orientations in the FE model, ensuring an accurate representation of the orientation histogram in the further analysis (Fig. 3a). Finally, the total density of the fibres (${\uprho }_{\text{f},\text{tot}}$) was set to 1.

To optimize computational efficiency, an analysis was performed to determine the minimum fibre count needed to accurately represent the global orientation in both ${\phi }$ and *θ* angles, while preserving model accuracy. The number of fibres for $\phi$ and *θ* were incrementally increased, with the resulting peak and equilibrium forces being tracked. The solution was deemed acceptable when the variation in forces upon adding fibres was less than 2%.

###  Local fibre orientation

To implement the local fibre orientation, the FE model of the tendon was first morphed to the experimental imaging data, after which the imaging data were used to define the fibre orientation histograms per element. Finally, the FE modelled with element-specific fibre orientation was morphed back to its original geometry.

Morphing was achieved by first downscaling the binarized imaging data by 50%; after that, morphological operators were used to obtain a solid, filled binarized volume. A triangulated mesh describing the outer shape of the tendon was created and used as the target for the morphing process. The morphing itself was performed by first registering the outer nodes of the FE model to the target geometry using a combination of scaling, rigid, affine, and nonlinear iterative closest point (Audenaert et al. 2019) registrations. Subsequently, the volumetric FE mesh was morphed to the registered outer shape using thin plate splines (Bookstein 1989). More details on this approach are available in Supplementary material S4.

After morphing, the voxels belonging to each element were identified, and an orientation histogram was created based on the fibre information from these voxels. The number of voxels within each element was on average ~ 44 000, with a range between 100 and 210.000. The fibre orientations for the FE model were then derived from the histogram in a similar fashion as the global orientation approach, with the same total fibre density, and transferred back to the corresponding elements in the unmorphed FE model (Fig. 3b).

###  Optimization

The material parameters for the fully loaded conditions were optimized to fit the experimental force data. Equal weight was set at the peak force, the fast relaxation region, and the equilibrium force (Supplementary material S5). The parameters were identified by trying to fulfil the constrains *h*_*i*_ based on the difference between simulation and experimental data (Eq. 8). The Method of Moving Asymptotes (MMA) optimization algorithm by Svanberg (1987) was used for solving the problem.

8 $${\text{subject to}}\;\;\begin{array}{*{20}c} {min 0} \\ {0 \ge - h_{i} } \\ {0 \ge h_{i} } \\ {h_{i} = {\varvec{F}}_{FE} \left( {t_{i} } \right) - {\varvec{F}}_{{\text{experiment }}} \left( {t_{i} } \right)\; \;\;t_{i} = 0 \ldots {\text{ end}}} \\ \end{array}$$

with ${t}_{i}$ representing the time at increment i, spanning from the start to the end of the simulation.

As collagen is the most load-bearing component, responsible for 95% of the total stresses in the tendon (Khayyeri et al. 2016), only the five parameters (${E}_{1}$, ${E}_{2}$, ${k}_{1}$, ${k}_{2}$, $\eta$) in the standard linear solid model were optimized, and the remaining parameters were kept constant, see Table 1. These parameters include k0, which is the initial permeability of the fluid flow and *M*_*k*_ which is a parameter to describe the nonlinear relationship between permeability and void ratio. Moreover, the model includes *E*_*p*_, *E*_*n*_, *ν*_*pn*_, *ν*_*n*_, and *G*_*pn*_, as the five independent material parameters of the transversely isotropic matrix, where *E* and *G* represent elastic and shear modulus, respectively, and subscripts *p*, *n*, and *pn* represent the in plane, normal, and transversal plane.

**Table 1:** Constitutive parameters that were kept constant for global, local, loaded, and unloaded models (Notermans et al. 2019). The optimized collagen parameters are available in Table 2

	*k*_*0*_ (mm/s)	*M*_*k*_(−)	*E*_*p*_ (MPa)	*E*_*n*_ (MPa)	*ν*_*pn*_ (−)	*ν*_*p*_ (−)	*G*_*pn*_ (MPa)
Value	1.43e−9	0.424	0.5	1	0.3	0.45	0.27

###  Evaluation of geometry and orientation

To determine the importance of geometry and fibre orientations on the difference in mechanical response between the FL and UL tendons, several simulations were performed using both FL model parameters:

- FLorient–FLgeom: simulation with FL orientation and FL geometry, optimized to the FL experimental data.
- FLorient–ULgeom: simulation with FL orientations but UL geometry.
- ULorient–ULgeom: simulation with both UL orientation and UL geometry.

This analysis was conducted for both global and local collagen orientation mapping with their respective optimized parameters. The aim was to evaluate how well adjustments in orientation and geometry could replicate the unloaded experimental conditions.

To further investigate the impact of fibre heterogeneity, we implemented straight fibres (elevation angle of 90 degrees and azimuth angle of 0 degrees), as in Notermans et al. (2019), for both FL and UL tendons as a baseline comparison. Together with the global and local orientations, this allowed for a detailed comparison of stress, strain, and orientation heterogeneity and highlighted the differences introduced by varying scales of fibre arrangement.

## Results

The minimal number of fibres required to accurately represent the global fibre orientation histograms was determined to be 7 ${\phi }$-angles and 5 *θ*-angles. Supplementary material S6 provides a more detailed explanation of the outcomes of this analysis. This configuration was applied in both global and local orientation analysis to ensure consistency across the scales. Subsequently, the material parameters of the constitutive model were optimized to the experimental data for the global and local fibre orientation separately using the FL geometry and orientation (Table 2).

**Table 2:** Optimized parameters to the FL global and local fibre orientations model

	*E*_*1*_ (MPa)	*E*_*2*_ (MPa)	*k*_*1*_	*k*_*2*_	*η*	RMSE
Global FL optimized	41.9	0.275	0.771	27.5	592	0.219
Local FL optimized	47.1	0.446	0.807	24.7	737	0.221

**Fig. 4:**
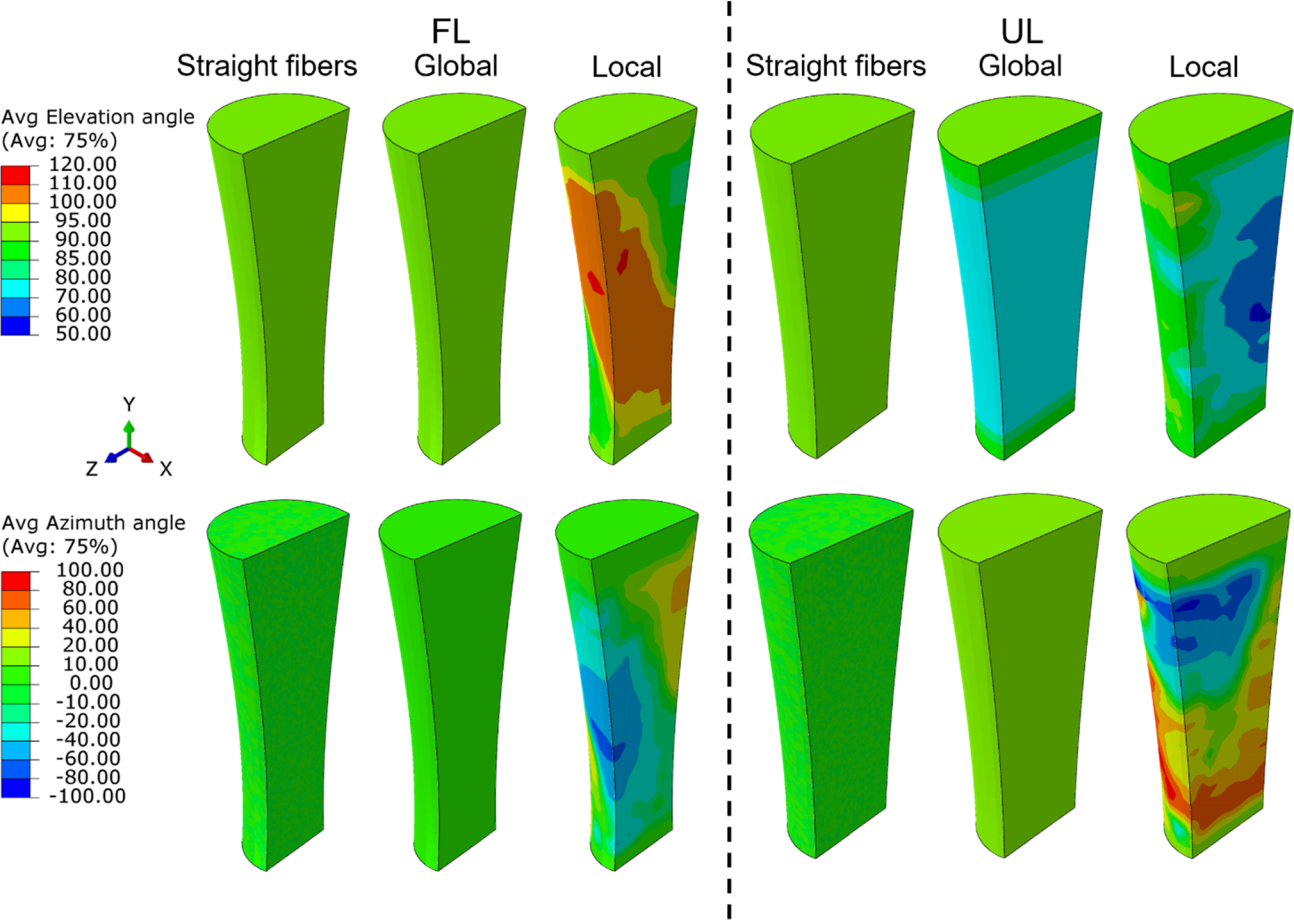
Fibre orientations for the fully loaded (FL) and unloaded (UL) tendon. The average elevation and azimuth angles are shown for the straight fibre, global fibre orientation, and local fibre orientation models

**Fig. 5:**
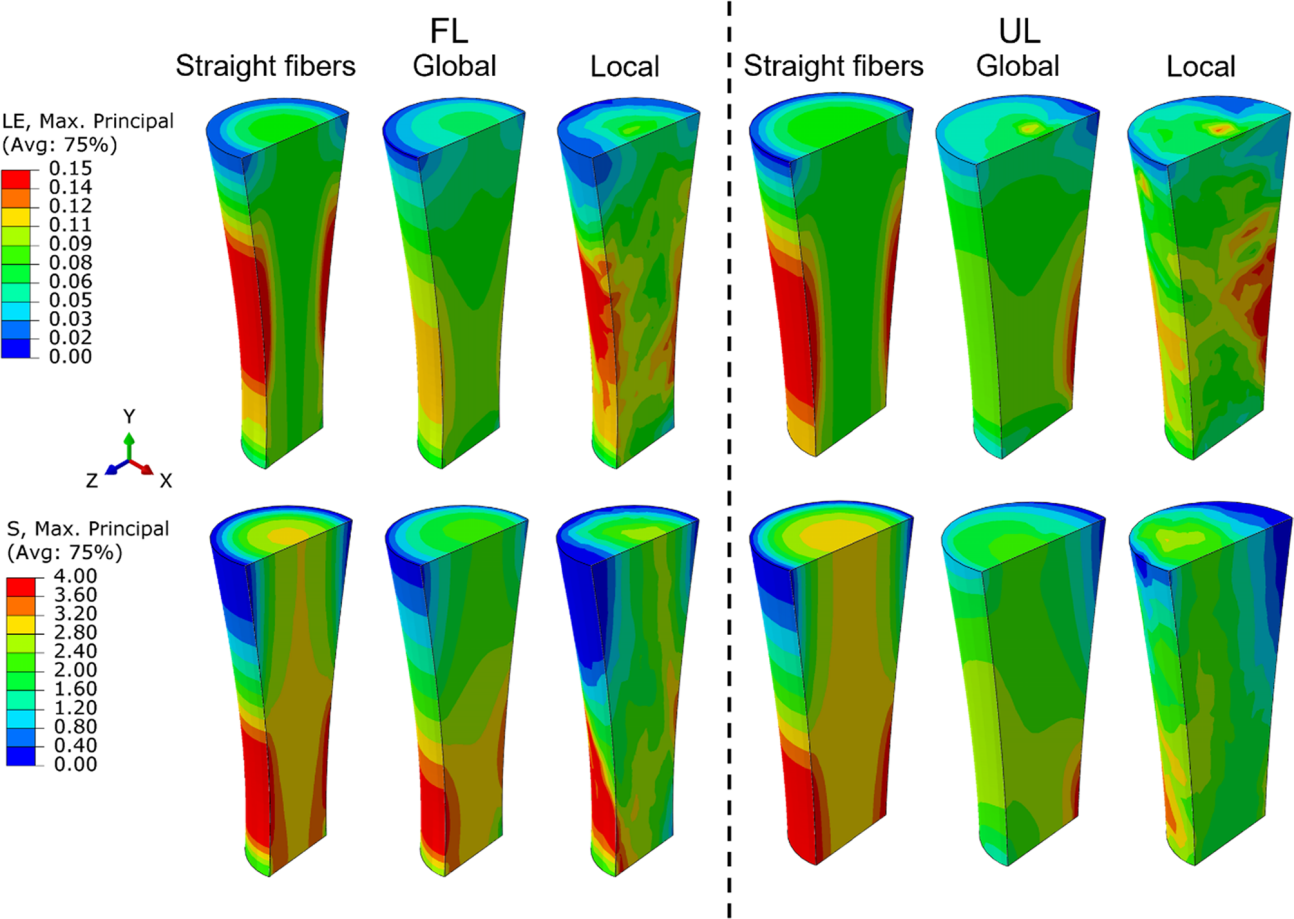
Maximum principal logarithmic strain (LE, top row) and maximum principal stress (S, bottom row) distributions within the FE models of the fully loaded (FL) and unloaded (UL) tendons, showing the variability between the straight fibre, global fibre orientation, and local fibre orientation models

The fibre orientation heterogeneity in the three models (straight–global–local) was demonstrated by calculating the average fibre orientation, determined as the mean of the 7 ${\phi }$-angles and 5 *θ*-angles used in the model’s orientation descriptions (Fig. 4). In this analysis, only the simulations with global optimized parameters are shown. The corresponding stress and strain distributions (Fig. 5) further illustrate the increased heterogeneity upon increased complexity of fibre orientation (straight–global–local).

**Fig. 6:**
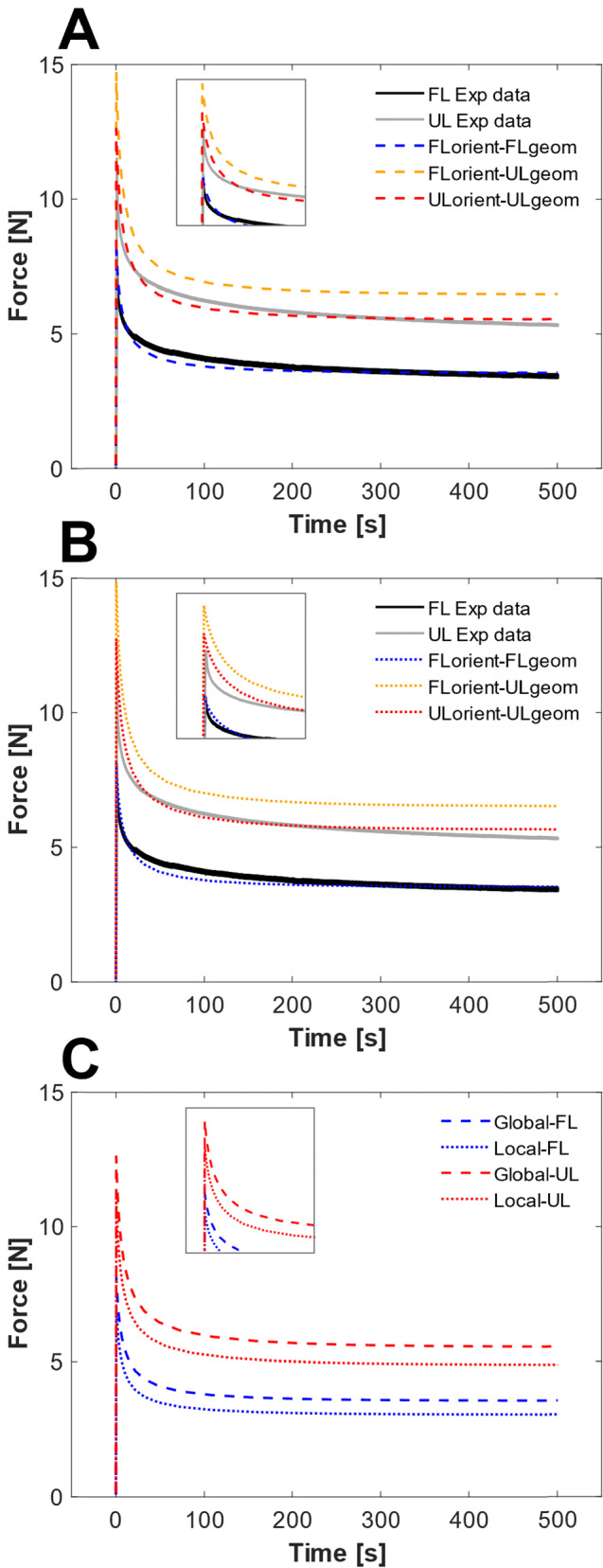
Force–time curves of the simulations. **A** Global orientation model simulations where the orientation and geometry are altered to the UL state, using the global optimized parameters. **B** Local orientation model simulations, using the local optimized parameters. **C** Comparison between global and local orientation models using the global optimized parameters

In terms of the force–time response (Fig. 6a–b and Table 3), the model with FL orientation and geometry (FLorient–FLgeom) closely aligned with the FL experimental data (RMSE of 0.25 for both global and local, Table 3) as the parameters were specifically optimized for this scenario. When the geometry was changed to the UL configuration while retaining the FL orientations (FLorient–ULgeom), the force was overestimated (RMSE of 1.05 and 1.12 for global and local, respectively, Table 3). However, incorporating the UL orientations alongside the UL geometry (ULorient–ULgeom) resulted in a relatively close match to the UL experimental data in both the global (RMSE of 0.36, Fig. 6a) and local (RMSE 0.38, Fig. 6b) models. The model parameters remained consistent within their respective global or local configurations, indicating that the combined adjustments of geometry and orientation successfully captured the observed changes in mechanical response between loading and unloading.

**Table 3:** Quantitative comparison of curves in Fig. 6 showing the percentage difference between experimental data and FE model at peak force (Peak%) and equilibrium force (Equil%), as well as the root-mean-square error (RMSE) between the modelled and experimental curve

	Global			Local		
	FLorient–FLgeom	FLorient–ULgeom	ULorient–ULgeom	FLorient–FLgeom	FLorient–ULgeom	ULorient–ULgeom
Peak%	1.4	28.0	9.6	0.3	29.4	10.9
Equil%	5.3	21.6	4.1	4.6	22.6	6.3
RMSE	0.25	1.05	0.36	0.25	1.12	0.38

Additionally, a comparison between global and local orientation analyses using the globally optimized parameters (Fig. 6c) revealed a reduced (~ -15%) force response for the local fibre orientation analysis. This may be attributed to the increased spatial heterogeneity in fibre orientations introduced in the local analysis. This additional heterogeneity disrupted the overall alignment of the fibres across the tendon, which led to a decreased collective force response.

## Discussion

This study advances our understanding of tendon adaptation to mechanical stimuli by integrating experimentally derived fibre orientation data into a computational model. Fibre orientations were analysed at both global and local length scales, where global orientations represented the average fibre alignment throughout the tendon, while the local approach captured the local fibre alignment and spatial heterogeneity. The investigation focused on whether tendon fibre orientation and geometry could explain the altered mechanical response observed in rat Achilles tendons after a period of unloading.

The findings showed that differences in fibre orientation and geometry explained the altered mechanical response between loading and unloading (Fig. 6). The ULgeom–ULorient case captured the UL experimental data (Table 3) while retaining the FL parameters. Additionally, in both global and local analysis, the percentage difference (Table 3) between FE model and experimental data at equilibrium force was approximately the same in the optimized FLgeom–FLorient case compared to the ULgeom–ULorient case, and the maximum difference at peak force (10%) fell within the variability of the experimental data (Pierantoni et al. 2023). This suggests that collagen fibres properties remained unchanged during unloading, aligning with Pierantoni et al. (2023), who found no notable difference in stiffness between loaded and unloaded tendons at the fibril (collagen) level. Rather than adjusting the model parameters to explain differences in mechanical response between loading and unloading, as proposed by Notermans et al. (2019), our study suggests that fibre reorientation and increased dispersion, combined with an increased tissue volume, is driving the altered mechanical behaviour. By introducing detailed fibre orientations, we thus eliminated the need for parameter adjustments, highlighting the critical role of fibre orientation in tendon mechanics and the limitations of overly simplified models.

When comparing the outcome of our global and local approaches, increased heterogeneity in fibre orientations resulted in an average 15% decreased force response (Fig. 6c), requiring adjustments in model material parameters (Table 2). This lower force response may be attributed to the less collective alignment of the fibres. However, the adjustment in material parameters did not alter the overall mechanical response between loading and unloading (Fig. 6b). Within the updated local orientation parameters, transitioning to the UL geometry and orientation still captured the unloaded mechanical response. The fact that the primary differences in mechanical response between UL and FL tendons can largely be attributed to fibre disorganization in UL tendons rather than variations in material properties aligns with the findings of Pierantoni et al. (2023), where no statistically significant differences in collagen type I, collagen type III, or elastin content between UL and FL tendons were reported. However, it is worth noting that UL tendons exhibited a decrease in cell density, which, on the long run, may impair their turnover capacity and, consequently, their mechanical properties.

Furthermore, the findings demonstrate that progressive incorporation of heterogeneity in fibre orientations increased the complexity of the mechanical response. While the average fibre orientation angles (Fig. 4) provided some insights into this heterogeneity, they did not fully capture the complexity from the 35 implemented angles as only a single average angle is shown. This complexity is more clearly reflected in the stress and strain distributions (Fig. 5), where each incremental step in fibre orientation (straight–global–local) introduced more pronounced localized patterns that cannot be captured with uniform fibre alignment assumptions. Notably, no clear direct relationship was found between the average orientation angles and stress or strain distributions (Fig. 4 and Fig. 5), likely because average fibre angles could not fully account for the nuanced influence of local heterogeneity on the mechanical response. Such localized stress and strain variations could be important considerations in tendon healing, cellular responses, and processes such as growth and remodelling.

The experimental data originated from a study where imaging and mechanical testing were performed separately and on separate animals. This was done to achieve the best possible image quality and control of the mechanical experiment. While combining these techniques in situ is possible when using phase contrast imaging with concurrent mechanical testing, it presents practical challenges. Our team have recently presented this approach on soft tissues (Dejea et al. 2024), including rat Achilles tendons (Pierantoni et al. 2025) which clearly visualized how the fibre distributions and orientations in the tissues are responding during in situ loading. However, in situ studies always come with compromises, most often resulting in reduced image quality (e.g. to keep imaging fast and radiation doses low) and non-dynamic or reduced loading speed. These challenges underscore the value of combining experimental data with computational models, as done in the present study, to overcome the inherent challenges of in situ imaging and enhance the accuracy of tendon structure analysis.

The current study presented a sample-specific approach to tendon fibre orientation modelling. By focusing on two representative tendons, the analysis captured sample-specific variability, which enhanced the understanding of the tendon structure. Ideally, the same tendons would have been used for both imaging and mechanical testing, but this was not feasible due to potential changes in mechanical properties after the high-resolution imaging (Pierantoni et al. 2023). Therefore, the average mechanical data were used instead (n = 9 for FL, n = 10 for UL). While averaging global orientations across multiple tendons might be feasible, averaging local orientations should be avoided as it can smooth out local variations, undermining the primary purpose of capturing localized fibre orientations and the full complexity of the tendon structure. Therefore, this sample-specific approach allows for effectively representing the individual tendon characteristics, making it valuable for detailed, tendon-specific modelling.

Preferably, our approach should be repeated on all experimentally imaged samples. However, as synchrotron imaging time is rare and scarce, most of the tendons in Pierantoni et al. (2023), were imaged in a smaller central field of view (4.2 × 3.5 mm) and not fully from the bone to muscle junction. Thus, we could not implement our approach on all tendons. Instead, to ensure repeatability, we kept the geometries constant and simulated the average fibre orientations from each group of samples in Pierantoni et al. (2023), using our global approach (not presented). The findings overall agreed well, lending credibility to our approach. However, it is worth pointing out that these were based on the average fibre distribution in a small central region and did not use the individual sample-specific geometry.

During periods of disuse, collagen becomes stiffer through increased cross-linking (Akeson et al. 1977). Maganaris et al. (2006) suggested that this might be counteracted by a reduction in longitudinally aligned collagen fibrils, which would decrease the overall stiffness. Our results support these suggestions since mechanical parameters did not change between loading and unloading. To deepen our understanding of how collagen stiffness evolves during unloading, future models could incorporate these cross-linking dynamics alongside additional fibre functionalities, such as recruitment and inter-fibre sliding. Although previous models have incorporated cross-links on the microscopic tendon level, they lack an adequate description of the tissue-level behaviour (Gautieri et al. 2013; Reese et al. 2013; Depalle et al. 2015). Incorporating fibre recruitment could follow approaches used for fibre reorientation during healing, growth, and remodelling (Driessen et al. 2003; Notermans et al. 2021b).

Furthermore, the currently employed method for the local orientation analysis was selected to maintain consistency with the global analysis, as both approaches generated histograms and defined the fibres from them. However, alternative methods could potentially be more computationally efficient, such as rotating the stiffness tensor according to principal fibre directions (Auenhammer et al. 2024). While the current model successfully captured the local fibre orientations, future research could compare various techniques for modelling local alignment, evaluating both accuracy and computational efficiency.

A limitation of this study is that only one mechanical test protocol from Pierantoni et al. (2023) was simulated. The main experimental loading condition (8% stress relaxation) was chosen to establish an initial comparison between loading and unloading using experimental fibre orientation data, without introducing excessive complexity. However, data from 16% stress relaxation and ramp-to-failure tests were also available from the same study (Pierantoni et al. 2023). Future efforts could aim to improve the current model’s robustness to accurately simulate additional loading scenarios. Additionally, the experimental data were obtained from rats following four weeks of unloading. While rats are valuable models for studying the effects of unloading, human tendons are characterized by greater complexity. For instance, they contain fascicles that can twist to redistribute stress within the tissue (Shim et al. 2018) and whose structural organization could also be possibly affected by unloading. Our study offers valuable insights on how tendon responses to unloading; nonetheless, translating these findings from rat models to human tendons requires caution. Further research is needed to address interspecies differences and enhance our overall understanding of tendon mechanobiology.

In conclusion, the current study implemented both global and local collagen fibre orientations into a computational framework to better understand tendon mechanics and, more specifically, explain whether the altered mechanical response from loading to unloading is caused by a change in mechanical properties or by altered fibre orientations. The findings indicated that orientation and geometry primarily account for the experimentally observed difference in mechanical response between loading and unloading, while material properties are kept constant. Additionally, introducing greater spatial heterogeneity reduced the overall force response but did not affect the changes in mechanical response between loading and unloading. The benefit of local heterogeneity can be important in future studies of mechanobiology of tendon healing, local cell responses, tissue growth or remodelling. Our study provides a comprehensive framework for exploring the mechanical behaviour of tendons under various conditions, highlighting the importance of considering fibre orientations in tendon constitutive modelling.

## Supplementary Information

Below is the link to the electronic supplementary material.

Supplementary file1 (GIF 3668 KB)

Supplementary file2 (PDF 468 KB)

Supplementary file3 (GIF 6686 KB)

## Data Availability

No datasets were generated or analysed during the current study.
