# Supplementary figures and images for: Investigating Achilles tendon adaptation to mechanical load: a computational model integrating collagen fibre orientation heterogeneity
Source: Biomech Model Mechanobiol. 2025 Aug 24;24(6):1959–72. doi: 10.1007/s10237-025-02002-0 (PMC12618352; doi:10.1007/s10237-025-02002-0)

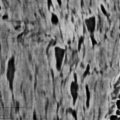

Supplement: Supplementary file 1 — Supplementary file1 (GIF 3668 KB) [file 10237_2025_2002_MOESM1_ESM.gif]

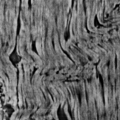

Supplement: Supplementary file 3 — Supplementary file3 (GIF 6686 KB) [file 10237_2025_2002_MOESM3_ESM.gif]
