# Supplementary material for: Investigating Achilles tendon adaptation to mechanical load: a computational model integrating collagen fibre orientation heterogeneity
Source: Biomech Model Mechanobiol. 2025 Aug 24;24(6):1959–72. doi: 10.1007/s10237-025-02002-0 (PMC12618352; doi:10.1007/s10237-025-02002-0)
Supplement: Supplementary file 2 — Supplementary file2 (PDF 468 KB) [file 10237_2025_2002_MOESM2_ESM.pdf]

SUPPLEMENTARY MATERIAL

INVESTIGATING ACHILLES TENDON ADAPTATION TO MECHANICAL LOAD: A COMPUTATIONAL MODEL INTEGRATING COLLAGEN FIBRE ORIENTATION HETEROGENEITY

Renate Janssen[1], Anna Gustafsson[1], Viktor Jönsson[1], Lorenzo Grassi[1], Maria Pierantoni[1], Hanna Isaksson[1]

1. Department of Biomedical Engineering, Lund University, Lund, Sweden

SUPPLEMENTARY MOVIES

Supplementary movie 1: The zoom in of the fiber structure in the fully loaded sample in Fig 2a is shown across a volume to exemplify fiber structural details.

Supplementary movie 2: The zoom in of the fiber structure in the unloaded sample in Fig 2a is shown across a volume to exemplify fiber structural details.

SUPPLEMENTARY METHODS

S1 Experimental details

The following details are from the study by Pierantoni et al. (2023). The data was reanalysed or reused in the current study.

<u>Synchrotron based phase contrast tomography</u>:
The tendons were stored at -20˚C and thawed about one hour before measurements were taken. The images were placed submerged in PBS during imaging. The imaging was performed at the X02DA TOMCAT beamline at the Swiss Light Source (SLS), Paul Scherrer Institute (Villigen, Switzerland) (Stampanoni et al. 2006), using a high numerical aperture microscope setup, 4x magnification, field of view (FOV) of 4.2 × 3.5 mm and a voxel size of 1.63×1.63×1.63 µm3. The propagation distance was 150 mm, and the X-ray energy was 15 keV. 2001 projections were acquired over 180° rotation. The exposure time was 33 ms resulting in about 90 s scans.

<u>Mechanical testing:</u>
Rat tendons were mechanically tested using an Instron 8511 load frame (Instron, USA) connected to an Interface SMT1-250 N load cell (Interface, USA), and controlled by MTS TestStarII (MTS Systems, USA). Tendons were thawed and their cross-sectional areas were measured. The muscle was carefully scraped off and the remaining tissue was clamped between sandpaper using flat grooved grips. The heel bone was clamped in a natural dorsiflexion configuration. The tendon tissue was kept hydrated during the test. After a 1 N preload, the tendons were loaded in tension. The testing protocol relevant for this study included a 1 N preload to straighten the tendon, followed by a pre-conditioning of cyclic loading (10 cycles, 6% strain, 0.1 mm/s), followed by 300 s of rest. Subsequently, a stress relaxation test of 8% and 16% strain was performed at 1 mm/s, each followed by 500 s relaxation time. The data from the 8% stress relaxation test is used in the current study.

S2 Cross sectional area analysis

The spatial area profile and the subsequently fitted second order polynomial (**Fig. *S1***) was used to align the cross-sectional area of the FE model from muscle to bone with the experimental cross-sectional area. The extended sections of the polynomial fit show how the cross-sectional area of the FE model is extended with 10% at the bottom and top.

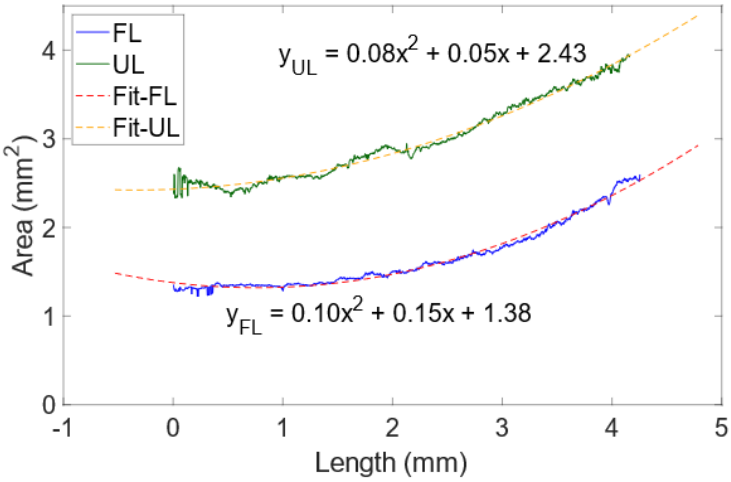

Fig. S1 Area profile over the length of the tendon, from bone to muscle insertion site. FL is the data from the fully loaded tendon and UL is the data from the unloaded tendon.

S3 Standard Linear Solids model

The consitutive description of the fibres consists of a standard linear solids description (***Fig. S2***) consisting of a spring in parallel with a Maxwell component. The spring represents the elastic behavior of the collagen network, while the Maxwell component captures the viscoelastic behavior.

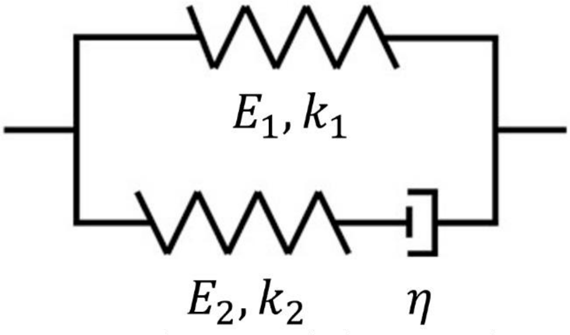

Fig. S2 Standard linear solids model.

S4 Mesh morphing

The image volume was first downscaled by 50% to reduce memory requirements. The cross-sectional slices were then dilated with structuring element size 20, followed by a filling of the holes and an erosion with the same element size. A tetrahedral mesh (**Fig. S3a**) was generated from the image volume using the iso2mesh toolbox (Qianqian Fang and Boas 2009) using default options except for the maximum surface element size of 20 and maximum deviation from segmented volume of 40. Subsequently, the mesh was aligned such that the midpoint of the x- and y-coordinates was centred at (0,0) (**Fig. S3b**) and the added top and bottom 10% of the FE mesh were removed so that both meshes had the same length (**Fig. S3c**). Morphing was performed by first registering the outer nodes of the FE model to the target geometry using a combination of scaling, rigid, affine and non-linear iterative closest point (Audenaert et al. 2019) registrations (**Fig. S3d**). Subsequently, the FE mesh was morphed to the reconstructed shape using thin plate splines (**Fig. S3e**) (Bookstein 1989).

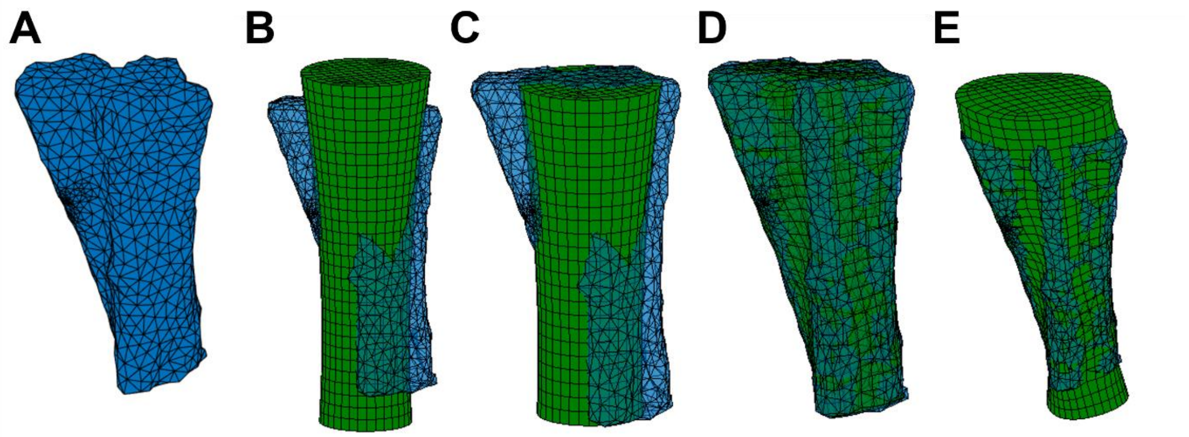

Fig. S3 Morphing steps, blue; mesh of image volume. green; finite element (FE) mesh. A) Tetrahedral mesh of the image volume. B) Alignment of meshes to the midpoint. C) Cropped FE mesh. D) Morphed FE mesh to the tetrahedral image mesh when FE model is still cropped. E) Morphed mesh used for voxel to FEM translation.

S5 Optimization of material properties

The points used for optimization (**Fig. S4**) were carefully chosen to ensure equal weight at the peak force, the fast relaxation region and the equilibrium force. These regions were given priority as they are the most important to capture the tendon's mechanical response.

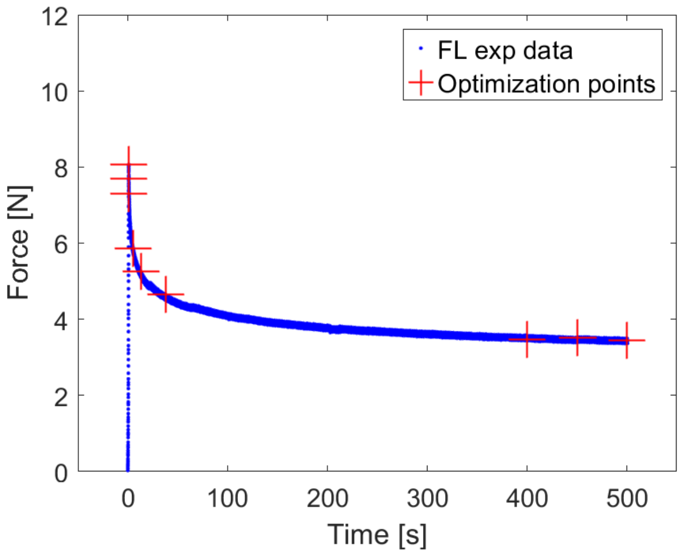

Fig. S4 Points used during optimization. Nine points were selected: three between the peak force and 80% of the peak force, three in the fast relaxation between 80% and 50% of the peak force, and three during the final relaxation phase, close to equilibrium.

S6 Number of fibres analysis

The fibre analysis conducted for the global fully loaded (FL) tendon determined the optimial number of fibres to be 7 ϕ-angles and 5 θ-angles based on the peak force analysis (**Fig. S5**). As increasing the total number of fibres from 35 (7 ϕ-angles and 5 θ-angles) to 48 (8 ϕ-angles and 6 θ-angles) would lead to a ~ 70% increase in computational time, we aimed to minimize the total number of fibres while still accurately representing the orientation histograms. The same analysis was conducted for the global unloaded (UL) tendon, resulting in 7 ϕ-angles and 4 θ-angles. As these numbers were equal or lower, we selected the FL ones to be consistent across all simulations. However, this approach was not extended to the local orientations, as each element had a unique histogram which required a different ideal number of fibres to describe its spread while maintaining computational efficiency. For consistency, the local analysis therefore used the same number of fibres as the global analysis.

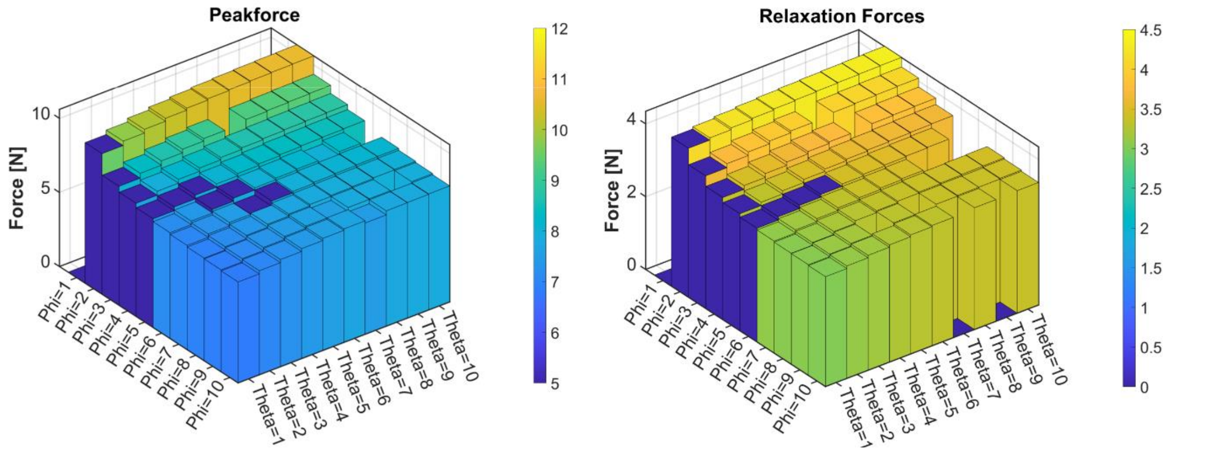

Fig. S5 Number of fibres analysis for the FL case. Each block represents the A) peak force or B) relaxation force for that simulation with corresponding number of ϕ and θ angles. The blue path illustrates the steepest descent trajectory, indicating the direction of the highest difference from the current value. The path terminates when a step would result in an increase of less than 2%.

REFERENCES

Audenaert EA, Van Houcke J, Almeida DF, et al (2019) Cascaded statistical shape model based segmentation of the full lower limb in CT. Comput Methods Biomech Biomed Engin 22:644–657. https://doi.org/10.1080/10255842.2019.1577828

Bookstein FL (1989) Principal warps: thin-plate splines and the decomposition of deformations. IEEE Trans Pattern Anal Mach Intell 11:567–585. https://doi.org/10.1109/34.24792

Pierantoni M, Silva Barreto I, Hammerman M, et al (2023) Multimodal and multiscale characterization reveals how tendon structure and mechanical response are altered by reduced loading. Acta Biomater 168:264–276. https://doi.org/10.1016/j.actbio.2023.07.021

Qianqian Fang, Boas DA (2009) Tetrahedral mesh generation from volumetric binary and grayscale images. In: 2009 IEEE International Symposium on Biomedical Imaging: From Nano to Macro. IEEE, pp 1142–1145
